# Mutations in genes related to myocyte contraction and ventricular septum development in non-syndromic tetralogy of Fallot

**DOI:** 10.3389/fcvm.2023.1249605

**Published:** 2023-09-28

**Authors:** Drayton C. Harvey, Riya Verma, Brandon Sedaghat, Brooke E. Hjelm, Sarah U. Morton, Jon G. Seidman, S. Ram Kumar

**Affiliations:** ^1^ Departments of Surgery, Keck School of Medicine, University of Southern California, Los Angeles, CA, United States; ^2^ Pathology, Keck School of Medicine, University of Southern California, Los Angeles, CA, United States; ^3^ Stem Cell Biology and Regenerative Medicine, Keck School of Medicine, University of Southern California, Los Angeles, CA, United States; ^4^ Department of Medicine, Rosalind Franklin University School of Medicine and Science, Chicago, IL, United States; ^5^ Translational Genomics, Keck School of Medicine, University of Southern California, Los Angeles, CA, United States; ^6^ Department of Pediatrics, Boston Children’s Hospital, Boston, MA, United States; ^7^ Department of Genetics, Harvard Medical School, Boston, MA, United States; ^8^ Pediatrics, Keck School of Medicine, University of Southern California, Los Angeles, CA, United States; ^9^ Department of Surgery, University of Nebraska Medical Center, Omaha, NE, United States

**Keywords:** tetralogy of fallot, exome sequencing, congenital heart defect, *de novo* variants, conotruncal defects

## Abstract

**Objective:**

Eighty percent of patients with a diagnosis of tetralogy of Fallot (TOF) do not have a known genetic etiology or syndrome. We sought to identify key molecular pathways and biological processes that are enriched in non-syndromic TOF, the most common form of cyanotic congenital heart disease, rather than single driver genes to elucidate the pathogenesis of this disease.

**Methods:**

We undertook exome sequencing of 362 probands with non-syndromic TOF and their parents within the Pediatric Cardiac Genomics Consortium (PCGC). We identified rare (minor allele frequency <1 × 10^−^4), *de novo* variants to ascertain pathways and processes affected in this population to better understand TOF pathogenesis. Pathways and biological processes enriched in the PCGC TOF cohort were compared to 317 controls without heart defects (and their parents) from the Simons Foundation Autism Research Initiative (SFARI).

**Results:**

A total of 120 variants in 117 genes were identified as most likely to be deleterious, with *CHD7*, *CLUH*, *UNC13C*, and *WASHC5* identified in two probands each. Gene ontology analyses of these variants using multiple bioinformatic tools demonstrated significant enrichment in processes including cell cycle progression, chromatin remodeling, myocyte contraction and calcium transport, and development of the ventricular septum and ventricle. There was also a significant enrichment of target genes of *SOX9*, which is critical in second heart field development and whose loss results in membranous ventricular septal defects related to disruption of the proximal outlet septum. None of these processes was significantly enriched in the SFARI control cohort.

**Conclusion:**

Innate molecular defects in cardiac progenitor cells and genes related to their viability and contractile function appear central to non-syndromic TOF pathogenesis. Future research utilizing our results is likely to have significant implications in stratification of TOF patients and delivery of personalized clinical care.

## 1. Introduction

Congenital heart disease (CHD) represents the most common birth defect, impacting roughly 1.8% of all live births globally, and is the leading non-infectious cause of infant mortality and pediatric hospitalization costs (1–4). Despite the devastating consequences and relatively high incidence, the etiology of most structural cardiac disease remains unknown. Tetralogy of Fallot (TOF), originally coined as “blue baby syndrome,” is the most prevalent form of cyanotic congenital heart disease (5%–10% of all CHD diagnoses, ∼3 per 10,000 live births) (5–8). TOF is defined by the combined presence of a sub-aortic ventricular septal defect, an overriding aorta, right ventricular hypertrophy and right ventricular outflow tract obstruction. TOF often requires corrective surgery during the first year of life, with a median total cost in the United States of America of $179,494 for primary repair or $222,799 for staged repair (9). While approximately 20% of cases of TOF are syndromic with an identified pathogenic gene, such as DiGeorge Syndrome (22q11.2 deletion), trisomy 21, Alagille Syndrome (*JAG1*), Ritscher-Schinzel-like Syndrome (*WASHC5*), and CHARGE Syndrome (*CHD7*) (10–14), 80% of cases are of unknown etiology (8). As the overall incidence and the increasing cost of care reduces accessibility to necessary treatments (15), it is imperative that the biological processes underlying aberrant heart development leading to TOF be defined.

Identification of the pathogenic origins of TOF have thus far focused on single driver genes, primarily by pedigree studies (7, 8). Conversely, animal model studies have elucidated important pathogenic pathways that may be driven by polygenic or environmental perturbations. There are several animal models of CHD, including conotruncal defects that present similar to TOF pathology observed in humans (16–20). Some of these models present with cardiac structural defects related to an environmental cause (i.e., prenatal alcohol exposure, maternal diabetes), while others are induced by genetic knockouts and haploinsufficiency, or a combination of both (21, 22, 23, 24, 25, 26). What is shared amongst these varied models is a disruption to either second heart field viability and proliferation, or cardiac neural crest cell viability and migration necessary for the maturation of the developing cardiac outflow tract. As such, a variety of perturbations in seemingly disparate genes and pathways may converge on the same biological processes and result in developmental derangements leading to TOF. For instance, regulation of secondary heart field cardioblast proliferation (GO:0003266; amigo.geneontology.org) has 60 associated genes. A deleterious mutation in any of the upstream genes in this process has the potential to result in TOF. Rare variants (MAF <0.01) with functional effects of transcripts involved in second heart field biology indeed were previously identified in a study of 93 non-syndromic TOF patients (27). We therefore hypothesize that deleterious genetic variants are enriched in specific pathways or biological processes among probands with non-syndromic TOF. Specifically, we hypothesize that TOF may be the result of impaired biological processes that are critical to progenitor cell development, mainly second heart field derived cells and cardiac neural crest cells which are the primary cell populations responsible for cardiac outflow tract development and contribute to development of the right ventricle and ventricular septum (28–30).

We set out to test this hypothesis by conducting *de novo* variant analysis on exome sequencing data of TOF patients collected and sequenced by the Pediatric Cardiac Genomics Consortium (PCGC). Our focus was on rare (minor allele frequency [MAF] of <1 × 10^−4^ according to Exome Aggregation Consortium) loss-of-function (LoF) and likely deleterious missense and non-frameshift indel (D-Miss/Indel) variants to maximize the likelihood of identifying variants with true pathogenic potential. We conducted a parallel *de novo* analysis of exome sequencing data from unaffected siblings who participated in the Simons Foundation Autism Research Initiative (SFARI) (i.e., those without autism spectrum disorder or heart defects/pathology) and their parents as a control group to evaluate the specificity of our TOF cohort enrichment results, as has been done in previous studies (31, 32). A variety of genetic analyses have been conducted utilizing the PCGC database studying *de novo* variants, single nucleotide variants, copy number variants, and indels in CHD more generally (i.e., after cohort aggregation of multiple heart defect phenotypes), and identified enrichment for cilia-related and chromatin-modifying genes (31, 33, 34). The study we performed here is unique by focusing solely on non-syndromic TOF probands, an approach that was recently successful in identifying novel candidate CHD genes in a study of non-rare, *de novo* variants in patients with TOF from the European Genome-phenome Archive (35).

## 2. Methods

### 2.1. Study population

#### 2.1.1. PCGC

The National Heart, Lung, and Blood Institute funded the PCGC and established the Congenital Heart Disease Genetic Network Study across six main and four ancillary recruitment sites across the United States of America and the United Kingdom (36). All participant members of the consortium provided informed consent. All cardiac diagnoses were made using an echocardiogram, other advanced imaging modality and operative reports. Information about the participant's demographics, family history, and additional data were collected through a combination of interviews and the participant's medical record. A total of 3,937 probands from PCGC were examined to select for cases with TOF (Supplementary Figures 1A, B). A total of 747 non-syndromic participants with TOF without a known chromosomal abnormality were identified. 404 had exome sequencing performed for the proband and both parents.

#### 2.1.2. SFARI

SFARI was started in 2003 to identify targets for research furthering the diagnosis and treatment of autism spectrum disorder (37). Within the many projects run by the foundation is the Simons Foundation Powering Autism Research (SPARK) initiative, which allows for use of its database for research outside of autism spectrum disorder (38). The initial SPARK cohort collected between 2016 and 2017 included 18,089 individuals with autism spectrum disorder and 28,515 family members. Trios were selected for this study to be comprised of an unaffected sibling and their parents, generating a proper control trio wherein the sibling proband has neither autism spectrum disorder diagnosis nor congenital heart disease. 350 randomly selected control trios from SFARI were analyzed.

### 2.2. Bioinformatic analysis

**Figure 1:**
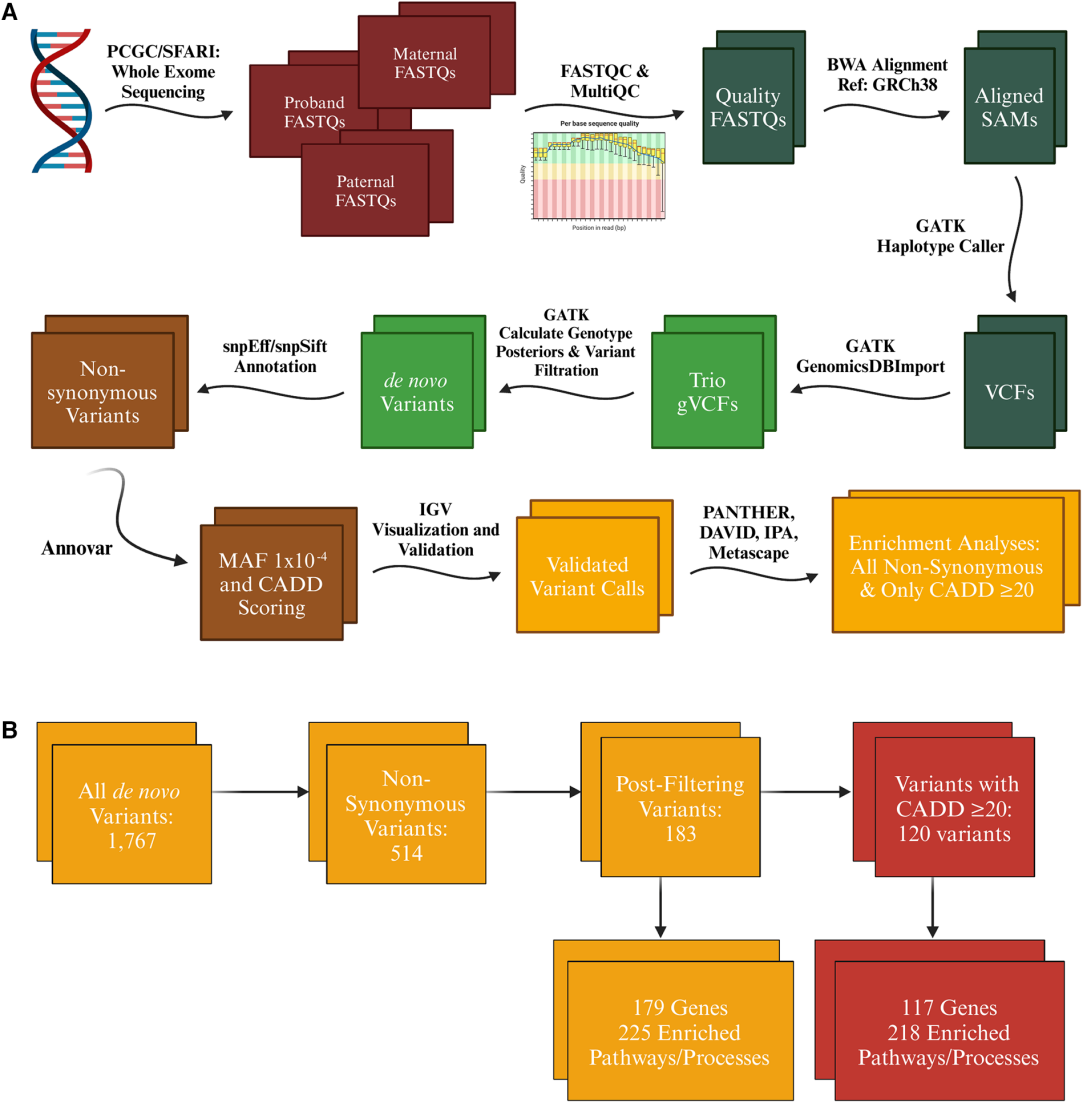
Variant call analysis of tetralogy of Fallot cohort. (**A**) Exome sequencing raw data was acquired from the Pediatric Cardiac Genomics Consortium (PCGC) for patients with non-syndromic tetralogy of Fallot (TOF) and Simons Foundation Autism Research Initiative (SFARI) for controls in the form of FASTQ and CRAM files respectively (which were converted to FASTQ format using samtools) for the proband and their associated parents. FASTQC and MultiQC were utilized to identify only high quality FASTQ files for analysis. All files were aligned to GRCh38 using BWA-MEM, then processed further according to GATK's best practices workflow until high confidence *de novo* variant calls were achieved for each trio. These variants were functionally annotated with snpEff, snpSift, and Annovar to identify variants that met our minor allele frequency threshold of 1 × 10^−4^ and were determined by snpEff (moderate or high impact) and the variant's CADD score (≥20) to likely be deleterious. (**B**) Initial variant calls included synonymous variants, which were first filtered down just to non-synonymous variants annotated as moderate or high impact by snpEff. Further filtering eliminated variants identified to be likely false positive calls and those with a minor allele frequency greater than 1 × 10^−4^. The number of variants was further reduced by filtering for only variants that had a defined CADD score of 20 or greater. A similar winnowing occurred with the SFARI cohort, however resulting in a smaller number of enriched pathways and processes in both the analysis of all non-synonymous variants (103) and only those with a CADD score of 20 or greater (59), all of which came from the Disease and Function analysis in IPA. * = Term enriched in both TOF and SFARI cohort. Figure generated with Biorender.com.

FASTQ files for all probands and their parents were assessed for quality control using FASTQC and MultiQC (Figure 1A) (39, 40). 90% of the FASTQ files for the selected TOF trios and 91% of SFARI control trios passed quality control (i.e., the proband and both parents passed) and were used for our analyses of *de novo* variants (PCGC TOF *n* = 362; SFARI control *n* = 317). Files were aligned with BWA-MEM to GRCh38 and then processed according to GATK Best Practices workflow (41, 42). Variants were called using GATK HaplotypeCaller. Variants were filtered for only high confidence *de novo* variants using GATK CalculateGenotypePosteriors, wherein both parents had a confident call for being homozygous for the reference allele. Functional annotation was next performed with snpEff and filtered with snpSift (43, 44). Additional functional annotation was conducted with ANNOVAR, including for MAF annotation derived from the Exome Aggregation Consortium (ExAC) and Combined Annotation-Dependent Depletion (CADD) scoring (45–47). CADD scores can score SNPs and indels based on their likelihood to be causal both on factors such as species conservation and impact on protein functionality. This analysis pipeline was automated utilizing Snakemake (48, 49). The complete bioinformatic pipeline for this analysis can be accessed *via* github^1^.

### 2.3. Variant filtering and enrichment analysis

Variants annotated as synonymous, upstream, downstream, 5′ or 3′ UTR, and intronic were filtered out (Figures 1A,B). Variants in genes known to be false positive calls in exome sequencing research were also filtered out (50). To focus on rare alleles exclusively we selected a MAF threshold of 1 × 10^−4^ according to ExAC via annotation by ANNOVAR. Only variants annotated by snpEff to be of moderate or high impact and a defined CADD phred score of 20 (representing approximately the top 1% of single nucleotide variants that had the highest prediction of functional effect) or greater were included in the enrichment analysis, with a secondary analysis conducted on all variants with a moderate or high impact annotation (snpEff) but no inclusion cutoff based on CADD phred scores.

A combination of Gene Ontology (GO) tools was used to determine enrichment of biological processes and pathways: the Protein ANalysis THrough Evolutionary Relationships (PANTHER) classification system^2^, the Database for Annotation, Visualization, and Integrated Discovery (DAVID) software^3^, Metascape^4^) and Qiagen's Ingenuity Pathway Analysis (IPA) software (51–55). The input to each of these tools was the variant list generated from our pipeline, one list of all *de novo*, non-synonymous variants and one list of only those with a defined CADD score of 20 or greater (Figure 1B). Genes identified in the variant analysis were evaluated for expression levels in the fetal heart via the Brotman Baty Institute's Atlas of Gene Expression During Development^5^ and those related to heart development were identified using IPA (56). All candidate variants were validated by visualization using Integrative Genomics Viewer (IGV) and for 65 probands that additionally had genome sequencing data available, variant calls from the exome sequence data were compared against variant calls from genome sequence data previously conducted by PCGC. None of the variants identified in the PCGC cohort was identified in the SFARI cohort, further supporting that effects seen are highly unlikely to be due to random enrichment of nonpathogenic alleles. Statistical analyses were conducted either by the GO analysis tools (PANTHER, DAVID, Metascape, IPA) or using the stats package in R (Version 4.0.2) (57).

### 2.4. Data availability

The data and study materials are under restricted access, as the data use agreements with PCGC and SFARI prohibit distribution of patient-level data, but upon application may be available to other researchers for the purposes of reproducibility or replicating the procedure. Data can be requested from both PCGC^6^ and SFARI^7^ by researchers. Exome sequencing data from PCGC was accessed via dbGaP authorized access of study phs001194.v3.p2 and exome sequencing data from SFARI was collected from the Simons Foundation Powering Autism Research for Knowledge (SPARK) project.

## 3. Results

### 3.1. Characteristics of study sample

Of the 362 patients in the final TOF cohort more individuals were assigned male at birth (*n* = 203) than female (*n* = 159; 56.08% vs. 43.92%). As these are just the cases of TOF that had exome sequencing data for the proband and both parents and the associated FASTQ files for all three passed initial quality metrics, this does not mean there is such a discrepancy amongst the total non-syndromic TOF population in the PCGC database. When looking at all non-syndromic conotruncal defects from PCGC however, a similar proportion was described with 57.6% described as male and 42.4% female (58). A similar breakdown was achieved in the SFARI cohort of 317 unaffected siblings of children with autism (53% male vs. 47% female). The racial and ethnic composition of the TOF cohort (Supplementary Figure 1A) is skewed more heavily white (83.1% vs. 72%), with the heaviest differences coming from far fewer Black individuals (5.8% vs. 13%) compared to the U.S. population at the time of sample collection and no individuals in our TOF cohort identified as American Indian, Alaska Native, Native Hawaiian or Pacific Islander. Unlike participants in PCGC's database, those in the SPARK project for SFARI did not have race or ethnicity data collected. Instead HapMap haplotypes were utilized to categorize ancestral background. The SFARI control cohort ancestral superclass composition was 3.42% African, 9.63% Admixed American, 1.24% East Asian, 79.81% European, 1.86% South Asian and 4.04% unknown ancestral superclass. While one cannot presume that the 79.81% EUR population is entirely white, the probability is high that the control cohort like the TOF cohort is predominately white.

### 3.2. Variant rate and characteristics of variants

The *de novo* mutation rate in the TOF cohort was 0.33 per proband for variants with a CADD score of 20 or greater (120 variants, 117 genes, 105 probands) and 0.56 per proband for all variants annotated as moderate or high impact by snpEff (183 variants, 179 genes, 147 probands; Supplementary Table 1). All variants identified after filtering are listed in Supplementary Table 1, with those with the 15 highest CADD scores listed in Table 1. The *de novo* mutation rate in our control cohort was 0.31 (97 variants, 97 genes, 82 probands) for those with a CADD score of 20 or greater and 0.51 overall (161 variants, 161 genes, 123 probands), neither of which was statistically different from the TOF cohort (Chi-square *p* = 0.88 and *p* = 0.47 respectively).

**Table 1:** *De novo* variants from tetralogy of Fallot cohort with highest CADD score.

Proband study ID	Gene	Fetal heart expression	snpEff impact; CADD score	Position	REF:ALT	AA change	Mutation	Enrichment
158	*EDNRA*	7%	High; 48	chr4: 147539934	C:T	p.Arg340*	Stop gained	**IPA:** Congenital anomaly of cardiovascular system, morphogenesis of blood vessel, Morphogenesis of cardiovascular system, perinatal death, transport of Ca^2+^, assembly of spindle apparatus, development of body axis, cell cycle progression, proliferation of neural cells, muscle contraction. **GO:** Aorta development, cardiac chamber development. **Disgnet:** Congenital heart defects, tetralogy of fallot, developmental disabilities, congenital arteriovenous malformation, neurodevelopmental disorders
322	*ABCC3*	0%	High; 42	chr17: 50684826	C:T	p.Gln1411*	Stop gained	
7	*GREB1l*	2%	High; 41	chr18: 21505458	G:A	p.Trp1373*	Stop gained	**IPA:** Formation of heart ventricle. **GO:** Cardiac chamber development. **Disgnet:** Congenital ear anomaly NOS (disorder).
35	*CHD7*	4%	High; 39	chr8: 60841997	C:T	p.Gln1599*	Stop gained	**IPA:** Congenital anomaly of cardiovascular system, Morphogenesis of blood vessel, morphogenesis of cardiovascular system, Familial central nervou system disease, Abnormality of inner ear, development of body axis, Cell cycle progression. **GO:** Aorta development, cardiac chamber development. **Disgnet:** Myopathy, congenital heart defects, congenital ear anomaly NOS (disorder), tetralogy of fallot, developmental disabilities, CHARGE syndrome, neurodevelopmental disorders.
7	*NAA15*	4%	High; 38	chr4: 139384958	C:A	p.Ser761*	Stop gained	**IPA:** Autism spectrum disorder or intellectual disability, familial central nervous system disease. **Disgnet:** Congenital heart defects, neurodevelopmental disorders.
188	*PIK3R4*	4%	High; 38	chr3: 130716473	G:A	p.Arg752*	Stop gained	**Disgnet:** Myopathy, congenital arteriovenous malformation, neurodevelopmental disorders
247	*COPB2*	4%	High; 38	chr3: 139373712	G:C	p.Ser283*	Stop gained	**IPA:** Organization of cytoskeleton
373	*CHD7*	4%	High; 38	chr8: 60838115	C:T	p.Arg1465*	Stop gained	
39	*SLC5A2*	0%	High; 37	chr16: 31486216	G:A	p.Trp172*	Stop gained	**IPA:** Congenital anomaly of cardiovascular system
212	*TNRC6B*	16%	High; 37	chr22: 40266712	C:T	p.Gln828*	Stop gained	**IPA:** Autism spectrum disorder or intellectual disability. **Disgnet:** neurodevelopmental disorders.
328	*CHAMP1*	1%	High;35	chr13: 114324530	C:T	p.Gln230*	Stop gained	**IPA:** Autism spectrum disorder or intellectual disability, familial central nervous system disease, organization of cytoskeleton.
138	*MAK16*	1%	Moderate; 33	chr8: 33489112	G:T	p.Arg122Leu	Missense variant	
76	*WASHC5*	13%	Moderate; 32	chr8: 125081679	A:T	p.Val167Asp	Missense variant	**IPA:** Familial central nervous system disease, assembly of spindle apparatus, cell cycle progression, organization of cytoskeleton. **Disgnet:** Myopathy, tetralogy of Fallot, CHARGE syndrome
12	*MED13l*	11%	Moderate; 32	chr12: 115963515	G:A	p.Ser2131Leu	Missense variant	**IPA:** Congenital anomaly of cardiovascular system, autism spectrum disorder or intellectual disability, familial central nervous system disease. **Disgnet:** Congenital heart defects, developmental disabilities, neurodevelopmental disorders.
169	*WAC*	3%	Moderate; 32	chr10: 28617658	C:T	p.Ala583Val	Missense variant	**IPA:** Autism spectrum disorder or intellectual disability. **Disgnet:** neurodevelopmental disorders.

Examining variants with a CADD score of 20 or greater in the PCGC TOF probands, 11.67% were LoF mutations and 88.33% D-Miss/Indel, compared to all variants where 14.75% were LoF and 85.25% D-Miss/Indel (Supplementary Table 2). By comparison, there were significantly fewer LoF variants with a CADD score of 20 or greater in the SFARI control cohort (Supplementary Figure 1C 4.12%, 4/97, OR 3.07, 95% CI 0.98–9.66, *p* = 0.05). There was no statistical difference in LoF variants between the two cohorts when examining all variants with moderate/high impact from snpEff alone (13.04%, 21/161, OR 1.15, 95% CI 0.62–2.13, *p* = 0.65). An evaluation of gene expression of candidate genes using the Brotman Baty Institute's Atlas demonstrated that more than half of all genes affected both in the CADD 20 analysis (50.43%, 59/117) and analysis of all variants (53.63%, 96/179) were found to be expressed in more than 1% of fetal cardiac tissue (56). This is significantly more than the number of genes affected in the control cohort, where only slightly over a third of genes were found to be expressed in the fetal heart in both analyses (CADD 20 analysis: 37.11%, 36/97, OR 1.72, 95% CI 0.99–2.98, *p* = 0.05; overall analysis: 36.02%, 58/161, OR 2.05, 95% CI 1.33–3.18, *p* = 0.001).

**Figure 2:**
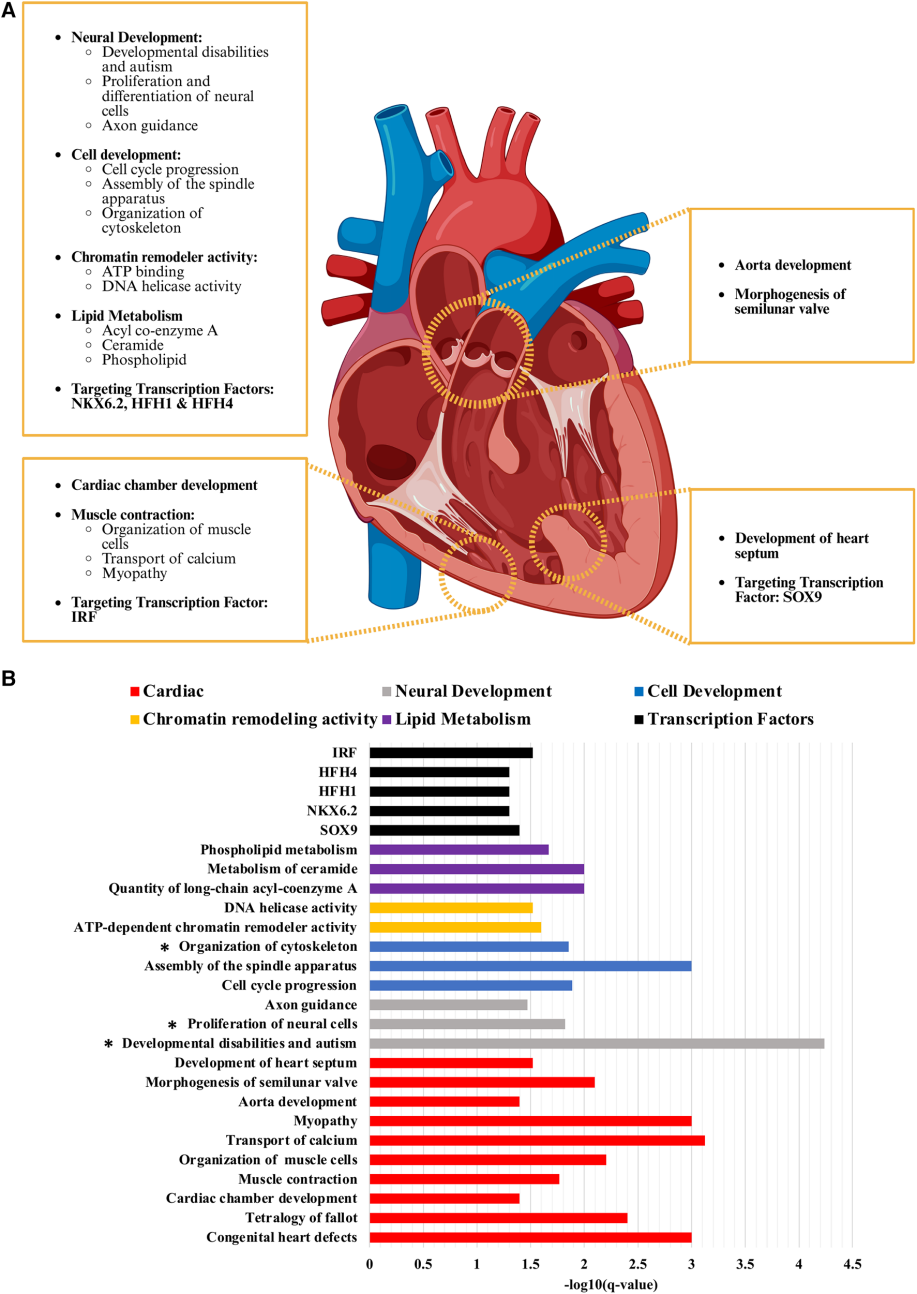
Critical pathways and biological processes enriched in non-syndromic tetralogy of Fallot. (**A**) While there are a variety of pathways and processes enriched in the analyses conducted on the *de novo* variants with a CADD score of 20 or greater identified from our cohort of non-syndromic tetralogy of Fallot, the most relevant can be considered in four main categories: those affecting the ventricle, the outflow tract, the ventricular septum, and extracardiac or more general pathways and processes (i.e. neural development, cell cycle progression). (**B**) The scale of how significantly enriched each term was determined by taking the negative log10 value of the B–H *p*-value (q-value) which has been corrected for multiple comparison testing. Cardiac related terms had an average -log10 of 2.19 ± 0.21 (S.E.M.), neural development 2.51 ± 0.87, cell development 2.25 ± 0.38, chromatin remodelling activity 1.56 ± 0.04, lipid metabolism 1.89 ± 0.11 and the transcription factor targets 1.36 ± 0.05. Figure generated with Biorender.com.

GO analysis found CHD (7-fold enrichment, *p* = 5.01 × 10^−7^, Benjamini-Hochberg (B–H) *p* = 0.003) to be enriched in our analysis, including TOF (8.5-fold enrichment, *p* = 1.26 × 10^−6^, B–H *p* = 0.004) and CHARGE syndrome (a syndromic form of TOF; 21-fold enrichment, *p* = 3.98 × 10^−5^, B–H *p* = 0.04; Figure 2, Table 2). Only CHD was significant in the analysis of all variants (5-fold enrichment, *p* = 6.31 × 10^−6^, B–H *p* = 0.02). According to IPA Disease and Function analysis there was also a significant overlap with genes involved in morphogenesis of the cardiovascular system (*p* = 1.94 × 10^−6^, B–H *p* = 5.69 × 10^−5^) and congenital anomaly of the cardiovascular system (*p* = 1.1 × 10^−6^, B–H *p* = 3.03 × 10^−5^). Overlap with these genes was less significant in the analysis of all variants (morphogenesis *p* = 8.57 × 10^−5^, B–H *p* = 1.67 × 10^−3^; anomaly *p* = 3.39 × 10^−4^, B–H = 5.85 × 10^−3^). The control cohort did not have overlap with any of these terms related to CHD or cardiac development.

**Table 2:** Enriched pathways and processes for tetralogy of Fallot cohort.

Description	GO Term	Count	Percentage of Variants	Enrichment	Raw *p*-value	B–H *p*-value
Metascape						
Enriched transcription factors						
SOX9 B1	N.A.	7	6%	7.6	3.98 × 10^−5^	0.04
NKX62 Q2	N.A.	7	6%	7.3	5.01 × 10^−5^	0.05
HFH1 01	N.A.	7	6%	7.2	6.31 × 10^−5^	0.05
AAAYWAACM HFH4 01	N.A.	7	6%	6.9	7.94 × 10^−5^	0.05
Enriched terms						
Aorta development	GO:0035904	5	4.27%	21.9	3.39 × 10^−6^	0.04
Cardiac chamber development	GO:0003205	7	5.98%	10.9	4.00 × 10^−6^	0.04
Disgnet						
Brachycephaly	C0221356	9	7.70%	12.0	5.01 × 10^−8^	0.001
Myopathy	C0026848	14	12%	5.7	1.58 × 10^−7^	0.001
Congenital heart defects	C0018798	11	9.40%	7	5.01 × 10^−7^	0.003
Congenital ear anomaly NOS (disorder)	C0266589	7	6%	13	1.00 × 10^−6^	0.004
Tetralogy of Fallot	C0039685	9	7.70%	8.5	1.26 × 10^−6^	0.004
Developmental disabilities	C0008073	10	8.50%	7.3	1.26 × 10^−6^	0.004
Postnatal growth retardation	C1859778	6	5.10%	13	7.94 × 10^−6^	0.02
End stage cardiac failure	C1868938	5	4.30%	17	1.26 × 10^−5^	0.02
CHARGE Syndrome	C0265354	4	3.40%	21	3.98 × 10^−5^	0.04
Congenital arteriovenous malformation	C0003857	6	5.10%	9.5	3.98 × 10^−5^	0.04
Neurodevelopmental disorders	C1535926	10	8.50%	4.8	5.01 × 10^−5^	0.04
Panther						
Cell development	GO:0048468	28	24%	2.78	6.19 × 10^−7^	0.01
ATP-dependent chromatin remodeler activity	GO:0140658	4	3%	19.61	7.54 × 10^−5^	0.025
DNA helicase Activity	GO:0003678	5	4%	11.59	9.62 × 10^−5^	0.03
ATP binding	GO:0005524	24	21%	2.75	5.61 × 10^−6^	0.005
David						
Membrane	GO:0016020	40	34.2%	1.9	3.3 × 10^−5^	0.008
EGF-like conserved site	IPR013032	5	4.30%	25.3	4.2 × 10^−5^	0.015
ATP binding	GO:0005524	23	19.7%	2.5	7.8 × 10^−5^	0.022
Ingenuity pathway analysis						
Congenital anomaly of cardiovascular system	N.A.	13	11%	N.A.	1.1 × 10^−6^	3.03 × 10^−5^
Morphogenesis of blood vessel	N.A.	7	6%	N.A.	1.94 × 10^−-6^	5.18 × 10^−5^
Morphogenesis of cardiovascular system	N.A.	11	9%	N.A.	2.15 × 10^−6^	5.69 × 10^−5^
Autism spectrum disorder or intellectual disability	N.A.	21	18%	N.A.	2.18 × 10^−6^	5.74 × 10^−5^
Familial syndromic hearing impairment	N.A.	5	4%	N.A.	5.93 × 10^−6^	1.42 × 10^−4^
Familial central nervous system disease	N.A.	32	27%	N.A.	6.14 × 10^−6^	1.46 × 10^−4^
Transport of Ca2+	N.A.	7	6%	N.A.	3.76 × 10^−5^	7.53 × 10^−4^
Abnormality of inner ear	N.A.	7	6%	N.A.	5.61 × 10^−5^	1.09 × 10^−3^
Assembly of spindle apparatus	N.A.	6	5%	N.A.	7.13 × 10^−5^	0.001
Perinatal death	N.A.	14	12%	N.A.	6.53 × 10^−5^	0.001
Development of body axis	N.A.	22	19%	N.A.	1.16 × 10^−4^	0.002
Adhesion of cell-associated matrix	N.A.	5	4%	N.A.	1.87 × 10^−4^	0.003
Organization of muscle cells	N.A.	4	3%	N.A.	4.09 × 10^−4^	0.006
Morphogenesis of semilunar valve	N.A.	3	3%	N.A.	5.3 × 10^−4^	0.008
Formation of heart ventricle	N.A.	5	4%	N.A.	5.7 × 10^−4^	0.008
Quantity of long-chain acyl-coenzyme A	N.A.	2	2%	N.A.	8.57 × 10^−4^	0.01
Metabolism of ceramide	N.A.	3	3%	N.A.	9.52 × 10^−4^	0.01
Cell cycle progression	N.A.	19	16%	N.A.	9.81 × 10^−4^	0.013
Organization of cytoskeleton	N.A.	25	21%	N.A.	0.001	0.014
Proliferation of neural cells	N.A.	14	12%	N.A.	0.001	0.015
Innervation of neurons	N.A.	4	3%	N.A.	0.001	0.016
Muscle contraction	N.A.	7	6%	N.A.	0.001	0.017
Growth of axons	N.A.	6	5%	N.A.	0.0015	0.018
Guidance of axons	N.A.	5	4%	N.A.	0.003	0.034
Development of heart septum	N.A.	4	3%	N.A.	0.004	0.03

A total of four genes were mutated in two probands in the TOF cohort: *CHD7*, *CLUH*, *UNC13C*, and *WASHC5* (Table 3). *CHD7* is a helicase critical in chromatin remodeling, *CLUH* is a mRNA binding protein involved in mitochondrial regulation, *UNC13C* is a membrane fusion protein involved in vesicle maturation and synaptic transmission and *WASHC5* is a component of the WASH complex that functions in endosomes (59–62). Variants in these genes were annotated as having a high impact by snpEff or an average CADD score greater than 20. No variant was identified in more than two probands. Of the genes mutated in two probands, *WASHC5*, *CLUH* and *CHD7* were identified to be expressed in the heart, of which *WASCH5* and *CHD7* have previously been identified to be related to CHD pathogenesis, including syndromic cases of TOF (8, 11, 14, 31, 63–65). Our findings further the case that these genes are also important in non-syndromic TOF. *CLUH* and *UNC13C* represent novel candidate genes that have not been previously associated with TOF pathogenesis, but are of high interest due to their observed perturbation in more than one unrelated probands.

**Table 3:** Genes mutated in more than one proband from tetralogy of Fallot cohort.

Probands Study IDs	Gene	Fetal heart expression	snpEff impact; avg CADD score	Position	AA change	Mutation	Enrichment
35, 373	*CHD7*	4%	High; 38.5	Chr8:60841997; 60838115	p.Gln1599*; p.Arg1465*	Stop gain	IPA: Congenital anomaly of cardiovascular system, morphogenesis of blood vessel, morphogenesis of cardiovascular system, familial central nervous system disease, abnormality of inner ear, development of body axis, cell cycle progression. GO: ATP binding, ATP-dependent chromatin remodeler activity, aorta development, cardiac chamber development, DNA helicase activity. Disgnet: Myopathy, congenital heart defects, congenital ear anomaly NOS (disorder), Tetralogy of Fallot, developmental disabilities, CHARGE syndrome, neurodevelopmental disorders
92, 171	*CLUH*	1%	Moderate; 28.65	chr17:2695241; 2701393	p.His823Tyr; p.Ala253Val	Missense	IPA: Perinatal death
76, 187	*WASHC5*	13%	High/ Moderate; 26.7	Chr8:125081679; 125056751	p.Val167Asp; p.Asp648Asn	Missense	IPA: Familial central nervous system disease, assembly of spindle apparatus, cell cycle progression, organization of cytoskeleton. GO: Cell development. Disgnet: Myopathy, tTetralogy of Fallot, CHARGE syndrome.
110, 370	*UNC13C*	0%	Moderate; 20.33	chr15:54264264; 54494714	p.Arg1182Gln; p.Asp1680Glu	Missense	

### 3.3. Confirmation of previous findings in tetralogy of Fallot genomic studies

The rate of likely damaging *de novo* variants (0.56) in our study is comparable to other studies of rare, *de novo* variants in cohorts of children with CHD (31, 66). Our analysis also identified variants in genes, pathways and biological processes previously identified in other studies of TOF. *CHD7*, *JAG1*, and *WASHC5* have been implicated in TOF, and *ATP2A2*, *CHD4*, *DNAH6*, *DTNA*, *EDNRA*, *FAT4*, *MED13l*, *RASA1*, and *SLC5A2* in CHD generally according to the literature and our IPA analysis (Supplementary Table 3) (8, 10, 11, 14, 35, 63, 64, 66, 67). A number of these genes have been established to be critical to the progenitor populations necessary for heart development: first and second heart field derived cells and cardiac neural crest cells. *EDNRA* is expressed for instance in the first heart field and *EDNRA* null mice have hypoplastic ventricles (68). *MED13l* is expressed in migratory cardiac neural crest cells, is heavily associated with transposition of the great arteries and coarctation and in cardiac energy metabolism and apoptosis (69). *FAT4* is expressed in cardiac mesoderm that produces both the first and second heart fields and has been demonstrated to regulate cardiac progenitor proliferation and planar cell polarity (70). Four genes had *de novo* variants that were found in multiple probands in our cohort, of which two (*CHD7* and *WASHC5*) are in genes previously implicated in TOF (Table 3). This finding speaks both to the centrality of these genes in TOF biology and the ability of our analysis to detect variants with high likelihood for pathogenicity.

Many exome sequencing studies of TOF have identified an enrichment for variants in genes related to autism, chromatin remodeling, cilia and ciliopathy, VEGF signaling, Notch signaling, and the Wnt/β-catenin pathway (71–77). Chromatin remodeler activity (19.61-fold enrichment, *p* = 7.54 × 10^−5^, B–H *p* = 0.025) was indeed found to be enriched in our cohort (Table 2), as was a significant relationship found for genes related to autism spectrum disorder and intellectual disability (*p* = 2.18 × 10^−6^, B–H *p* = 5.74 × 10^−5^). It is worthy to note that autism spectrum disorder and intellectual disability (*p* = 0.003, B–H *p* = 0.03) was also found to be enriched in our control cohort though to a lesser degree. This may be in relation to the large number of genes encompassed in this disease (SFARI's Gene database lists 942 genes as of 2022) and a reflection of the disagreement between datasets and research groups on what true risk genes are for autism spectrum disorder and intellectual disability (78–80). Ciliary body morphogenesis (GO:0061073; 42.89-fold enrichment, *p* = 0.03, B–H *p* = 1.00), the Notch signaling pathway (P00045; 7.63-fold enrichment, *p* = 0.03, B–H *p* = 0.81) and the Wnt signaling pathway (P00057; 3.82-fold enrichment, *p* = 0.003, B–H *p* = 0.43) were found to be enriched in the variants called in our TOF cohort; however, these pathways were not significant after correction for multiple comparison testing.

### 3.4. Pathways and biological processes identified with pathogenic potential

Molecular function GO analysis demonstrated that ATP binding (2.75-fold enrichment, *p* = 5.61 × 10^−6^, B–H *p* = 0.005) and DNA helicase activity (11.59-fold enrichment, *p* = 9.62 × 10^−5^, B–H *p* = 0.03) were found to be enriched in our identified variants with a CADD score of 20 or greater (Figure 2, Table 2). Both terms were also significantly enriched in the analysis of all variants, in addition to meiotic cell cycle (GO:0051321; 6.83-fold enrichment, *p* = 2.40 × 10^−6^, B–H *p* = 0.05). Biological function GO analysis elicited that aorta development (21.9-fold enrichment, *p* = 3.39 × 10^−6^, B–H *p* = 0.04), cardiac chamber development (10.9-fold enrichment, *p* = 4.00 × 10^−6^, B–H *p* = 0.04), and general cell development (2.78-fold enrichment, *p* = 6.19 × 10^−7^, B–H *p* = 0.01) were significantly enriched. By comparison, no functional GO terms were significantly enriched in the analysis of all variants. Diseases identified to be enriched related to our variants are myopathy (5.7-fold enrichment, *p* = 1.58 × 10^−7^, B–H *p* = 0.001), congenital ear anomaly (13-fold enrichment, *p* = 1.00 × 10^−6^, B–H *p* = 0.004) and developmental disabilities (7.3-fold enrichment, *p* = 1.26 × 10^−6^, B–H *p* = 0.004).

IPA analysis detected a significant overlap with genes involved in cell development, neural development, muscle development and function, lipid metabolism, hearing impairment (*p* = 5.93 × 10^−6^, B–H *p* = 1.42 × 10^−4^), perinatal death (*p* = 6.53 × 10^−5^, B–H *p* = 0.001), development of the body axis (*p* = 1.16 × 10^−4^, B–H *p* = 0.002), morphogenesis of semilunar valve (*p* = 5.3 × 10^−4^, B–H *p* = 0.008), and development of the heart septum (*p* = 0.003, B–H *p* = 0.03). Processes involved in cell development include assembly of the spindle apparatus (*p* = 7.13 × 10^−5^, B–H *p* = 0.001), adhesion of cell-associated matrix (*p* = 1.87 × 10^−4^, B–H *p* = 0.003), cell cycle progression (*p* = 9.81 × 10^−4^, B–H *p* = 0.013), and organization of cytoskeleton (*p* = 0.001, B–H *p* = 0.014). Included in neurocognitive development are familial central nervous system disease (*p* = 6.14 × 10^−6^, B–H *p* = 1.46 × 10^−4^), proliferation of neural cells (*p* = 0.001, B–H *p* = 0.015), innervation of neurons (*p* = 0.001, B–H *p* = 0.016), growth of axons (*p* = 0.0015, B–H *p* = 0.018), and guidance of axons (*p* = 0.003, B–H *p* = 0.034). The analysis of all variants revealed an additional enrichment for differentiation of neural cells (*p* = 6.95 × 10^−4^, B–H *p* = 0.05). Related to muscle development and function are transport of calcium (*p* = 3.76 × 10^−5^, B–H *p* = 7.53 × 10^−4^), organization of muscle cells (*p* = 4.09 × 10^−4^, B–H *p* = 0.006), and muscle contraction (*p* = 0.001, B–H *p* = 0.017). We have grouped calcium transport into the category of muscle development and function as we believe that it is distinctly tied to dysfunction of the sarcoplasmic reticulum. *ATP2A2* and *RYR1* are shared between calcium transport, muscle contraction and organization of muscle, with *ANXA6* and *EDNRA* additionally shared between calcium transport and muscle contraction. While not significant after multiple comparison testing, both calcium regulation in cardiac cells (WP536; 8.49-fold enrichment, *p* = 3.24 × 10^–4^, B–H *p* = 0.25) and sarcoplasmic reticulum calcium ion transport (GO:0070296; 22.88-fold enrichment, *p* = 0.004, B–H *p* = 0.65) specifically were enriched. Related to lipid metabolism, quantity of long-chain acyl-coenzyme A (*p* = 8.57 × 10^−4^, B–H *p* = 0.01) and metabolism of ceramide (*p* = 9.52 × 10^−4^, B–H *p* = 0.01) were found to be significant, with the additional terms of accumulation of phosphatidylinositol 3,4-diphosphate (*p* = 0.001, B–H *p* = 0.02), accumulation of phospholipid (*p* = 0.002, B–H *p* = 0.02), and accumulation of phosphoinositide (*p* = 0.004, B–H *p* = 0.05) significantly enriched in the analysis of all variants. A majority of the enrichments from the CADD 20 analysis were found in the IPA analysis of all variants (Supplementary Table 3). Of note, terms associated with calcium handling and muscle contraction were only identified in the CADD 20 analysis (concentration of Ca^+2^, liberation of Ca^+2^, oscillation of Ca^+2^, transmembrane transport of Ca^+2^, formation of muscle, hereditary myopathy, and muscle contraction; *p*-values and B–H values in Supplementary Table 3), as was development of heart septum, demonstrating the critical nature of the CADD 20 analysis.

Of final note, there was also significant enrichment of target genes of four different transcription factors, all of which have previously been tied to cardiac development or injury response. The most significant is *SOX9* (7.6-fold enrichment, *p* = 3.98 × 10^−5^, B–H *p* = 0.04), which coincidentally is the only transcription factor that has been previously reported in relation to TOF and is critical for ventricular septum formation (81, 82). *NKX6*.*2* (7.3-fold enrichment, *p* = 5.01 × 10^−5^, B–H *p* = 0.05) has been previously tied to CHD. While no specific role of *NKX6.2* in cardiac development has been identified, the Nkx family (particularly *NKX2*.*5*) plays a critical role in specification of the second heart field pharyngeal mesoderm (83, 84). *HFH1* (7.2-fold enrichment, *p* = 6.31 × 10^−5^, B–H *p* = 0.05), also known as *FOXQ1*, is involved in cell proliferation, differentiation, and myocardial fibrosis, though it has mainly been studied in cancer (85, 86). *HFH4* (6.9-fold enrichment, *p* = 7.94 × 10^−5^, B–H *p* = 0.05) has been implicated in ciliogenesis. This process by itself is not enriched in our TOF cohort but has been reported previously in other genomic analyses of TOF and left-right body axis formation, which we demonstrated is enriched in our cohort (34, 87). In contrast, none of the four transcription factors enriched in the CADD 20 variant list is significant after multiple comparison correction in the overall list. This again demonstrates the importance of the CADD 20 analysis in identifying the variants and their associated pathways that would not be elucidated from the analysis of all *de novo* non-synonymous variants in the cohort. *IRF* is the only transcription factor of significance (6.2-fold enrichment, *p* = 1.58 × 10^−5^, B–H *p* = 0.03). *IRF* is enriched in the CADD 20 analysis (5.3-fold enrichment, *p* = 0.003, B–H *p* = 0.46), though not after correction for multiple comparison testing. *IRF* is predominately connected to cardiac biology through its role in cardiac fibrosis, ventricular remodeling, and heart failure (88, 89, 90). The fact it is significantly enriched in the larger analysis, that it has a functional similarity to *HFH1*/*4*, and the stringency of our various filtering metrics warrants further consideration of its role in TOF pathogenesis and prognosis.

Enrichment analyses of the control cohort resulted in no identified enriched pathways outside of analysis with IPA. The only overlap in the IPA analysis was for enrichment in growth of axons (*p* = 5.47 × 10^−4^, B–H *p* = 0.009), proliferation of neural cells (*p* = 0.002, B–H *p* = 0.02), organization of cytoskeleton (*p* = 0.005, B–H *p* = 0.04), and autism spectrum disorder or intellectual disability (*p* = 0.003, B–H *p* = 0.03). While growth of axons was more significantly enriched than in the TOF cohort, all other terms were enriched to a lesser degree than the TOF cohort. Whether or not these should not be considered as potentially implicated in TOF pathogenesis requires further study.

## 4. Discussion

This work is the largest exome sequencing rare, *de novo* variant analysis to date focused exclusively on non-syndromic TOF, a critical step to addressing the yet unknown pathogenesis underlying the most prevalent cyanotic heart defect. Our analysis revealed a significant enrichment of variants in pathways involved in aortic and cardiac chamber development, and CHD, including a specific enrichment for TOF. Further, a high percentage of deleterious variants were observed in genes expressed in the fetal heart. Similar to prior studies, our work also identified the importance of chromatin remodeling genes in cardiac development and overlap with genes involved in autism spectrum disorder and neurodevelopmental disorders (71, 75).

While this study is focused on the crucial nature of pathways and processes found to be significantly enriched in our TOF cohort to elucidate knowledge about its etiology, the potential critical nature of the four genes found to be mutated in multiple probands should not be ignored. *CHD7* is involved in cardiac development via neural crest cells for which it has been described as critical for their migration, a critical function related to their role in cardiac outflow tract development (59, 91, 92). *CHD7* also plays a critical role in the transition of cardiac specified mesoderm becoming the second heart field, including sharing numerous enhancer sites with the critical second heart field gene *ISL1* (93). The second heart field importantly is the primary progenitor population responsible for outflow tract development. *WASHC5*, previously known as *KIAA0196*, is responsible for production of strumpellin and has been implicated not only in heart development, but also in motor neuron function (62). *CLUH* and *UNC13C* represent novel, putative candidate genes involved in TOF pathogenesis. *CLUH* is an RNA binding protein critical in regulating mitochondrial fission and both oxidative phosphorylation and fatty acid metabolism in the mitochondria (60, 94). Cardiomyocyte development relies on a switch from dependence on glycolysis to oxidative phosphorylation during embryonic development and eventually fatty acid oxidation during late fetal and postnatal development, a lack of which is incompatible with cardiomyocyte differentiation and maturation (95–97). A cessation of mitochondrial fission is additionally required for cardiomyocyte differentiation (98). The critical nature of these changes (demonstrated both in rodent models and stem cell models of cardiac development) may explain the potential role for *CLUH* in TOF pathogenesis, as a lack of cardiac differentiation would explain the structural defects that define TOF (99). An alternative hypothesis is that mitophagy has also been demonstrated to begin upon differentiation of stem cells to cardiac progenitors and is necessary for proper mitochondrial network formation, cellular stress response and cell survival (100). Knockdown of *CLUH* has been demonstrated to cause a block of mitophagy (101). While *CLUH* knockout mice have been reported to have phenotypically normal hearts, the knockout was associated with neonatal lethality (94). *UNC13C* is involved in membrane fusion and is a central player in synaptic transmission processes, including calcium coupling (61). *UNC13C*'s centrality in synaptic transmission adds to the evidence of a potential link directly between CHD and nervous system development and autism which has also been associated with synaptic dysfunction (102).

We believe that the critical underlying thread shared throughout the pathways and biological processes found to be significantly enriched in our cohort is a vulnerability related to cell cycle progression, differentiation and typical organization that prohibits formation of the ventricular septum and proper function of the ventricle. Animal models of tetralogy-type double outlet right ventricle (DORV) provide mechanistic insights into the role of progenitor cell proliferation and organization in this defect (19, 20, 103). When there is an inadequate proliferation of second heart field and cardiac neural crest progenitor cells, there is a reduction in the pool of cells available to incorporate into and elongate the developing cardiac outflow tract, resulting in improper alignment (104, 105). A foreshortened outflow tract, unable to appropriately align over the two ventricles, results in TOF-type defects (19, 20). Cellular organization is also crucial for elongation and, more importantly, appropriate rotation of the developing outflow tract. Early results in these animal models indicate a close interaction between neural crest cells and second heart field progenitors that regulates polarized migration of cells into the outflow tract for its appropriate elongation and rotation (103). Our human data in the current study supports this hypothesis as indicated by significant overlap of pathways associated with cell proliferation and with genes necessary for normal ventricle, outflow tract and myocyte development that are expressed in the first and second heart field, such as EDNRA and FAT4, and migratory cardiac neural crest cells, such as *MED13l*. In this regard, the enrichment for target genes of *SOX9* is particularly intriguing, as loss of *SOX9* has been demonstrated to result in TOF in murine models *via* a loss of second heart field contribution to outflow tract mesenchyme resulting in a perturbation of the proximal outflow tract septum and an associated membranous ventricular septal defect (81). This loss of *SOX9* was also associated with cleft palate in addition to skeletal malformations and sex reversal. We have shown that the most common heart defect over-represented in children with cleft disease is TOF (106). Furthermore, in our focused genome analysis of children with concomitant cleft and outflow tract congenital heart defect, we identified a *de novo* mutation in the *MED12* gene as likely pathogenic (107), and this gene is known to interact with *SOX9*. Finally, in relation to the importance of second heart field biology, a potential link may exist with the enrichment found in lipid metabolism. While deficiencies in maternal phospholipid and fatty acid metabolism have been associated with CHD, with potential implications specifically for maternal diabetes related CHD, a direct relationship between fetal lipid metabolism and cardiac development has not yet been established (108). Cell membrane lipid composition and lipid metabolism, however, have been demonstrated to be of critical importance in pathways critical both to second heart field development and TOF pathogenesis, Notch and Wnt signaling (109–113).

Our analysis also identified variants that impacted processes related to sarcoplasmic reticulum dysfunction (muscle cell organization, calcium handling, and contraction) and thereby, may impact ventricular function. A prior study of targeted sequencing of 22 families with non-syndromic TOF probands also identified sarcomere dysfunction as enriched in their cohort (72). Only one shared gene (*MYOM2*) was affected in our cohort and theirs. Such an overlap has been previously observed in the pathogenesis of nemaline myopathy in skeletal muscle, where there is association between myopathy, sarcomere function, and contractile deficits (114). However, a defect in cardiomyocyte contraction and sarcomere function is particularly interesting in the context of care of patients with TOF. Following complete surgical repair of TOF, a subset of patients develops right ventricular dilation and dysfunction over time. It would therefore be interesting if there is correlation between these clinical outcomes and any of the genetic abnormalities identified in our analysis. Early identification of patients with deleterious variants in pathways related to ventricular function could allow for more personalized clinical care approaches in the future.

As in other genetic studies of CHD, this study demonstrates an overlap between genes involved in autism spectrum disorder and intellectual disabilities and TOF (71, 75). The basis for this overlap is unclear, and additional research is required to understand if there are shared molecular pathways between organization of myocyte and axon cytoarchitecture. There appears to be a significant enrichment for variants involved in neural cell progenitor proliferation and differentiation. In addition to the effects these genes/variants may have in brain development, it is an intriguing possibility that these may also impact cardiac neural crest cells and may be of relevance in heart development. Lastly, what effect these genetic variants in neurons have on neurologic outcomes in children with TOF has important clinical implications. This is particularly relevant for a disease like TOF, where cyanosis is ubiquitous in early infancy and surgical repair requires the use of cardiopulmonary bypass with its attendant adverse effects on the developing nervous system.

There are distinct limitations to our study in relation to its demographic composition and overall size. The demographics of our study cohort, like most studies in biomedical research, were skewed toward a white population, including no reported American Indian, Alaska Native, Native Hawaiian or Pacific Islander individuals. Due to documented methodologic inadequacies and structural barriers rooted in white, settler colonialism, Indigenous patients may be a part of our cohort but are simply not identified as such, as has been observed in documenting the incidence of other diseases such as COVID-19 (115, 116). This is less concerning to this analysis given its focus on *de novo* variants rather than germline mutations. However, continued work remains necessary to include populations most disproportionately impacted by CHD-associated morbidity and mortality in future studies (117, 118). Additionally, while there appears to be a higher number of individuals assigned male at birth in our TOF cohort than female, we are unable to make any inference from our results as to a potential sex bias in the pathology due to the filtering of individuals in the PCGC database with non-syndromic TOF that did not have exome sequencing or if one or both of their parents lacked exome sequencing. There have been conflicting reports as to whether TOF is more common amongst those assigned male at birth or if there is no significant sex-based difference (58, 119–122).

Although ours is the largest non-syndromic TOF exome sequencing study of *de novo* variants to date, the sample size is still limited and precludes more extensive enrichment analyses. Given the high statistical threshold required to reach significance when correcting for multiple comparison testing in pathway enrichment analysis, it is important to note that there are some processes that were identified as being enriched but not significant in our analyses after this correction. Some of these processes may still have biological relevance, as evidenced by the fact the Wnt and Notch signaling pathways have previously been identified in animal models as being associated with TOF pathology and were enriched in our study, yet neither was significant after correcting for multiple comparisons. Even with a limited sample size, we were still able to identify both novel candidate genes and pathways and processes as has been done in other limited exome sequencing studies with fewer trios than our TOF cohort (123, 124).

## 5. Conclusion

In summary, using exome sequencing in a cohort of 362 non-syndromic TOF patients, we demonstrate that innate molecular defects in cardiac progenitor cells related to their viability and contractile function play an important role in TOF pathogenesis. Whether and how these genetic variants impact clinical outcomes in these patients requires more focused analyses that correlate variants in these pathways to clinical outcomes and long-term complications. The critical next step is to utilize this work to experimentally study disruption of the identified pathways and biological processes, with particular attention to the genes mutated in our cohort.

## Data Availability

The data analyzed in this study is subject to the following licenses/restrictions: The data and study materials are under restricted access, as the data use agreements with PCGC and SFARI prohibit distribution of patient-level data, but upon application may be available to other researchers for the purposes of reproducibility or replicating the procedure. Requests to access these datasets should be directed to PCGC, https://www.ncbi.nlm.nih.gov/projects/gap/cgi-bin/study.cgi?study_id=phs000571.v6.p2. SFARI, https://www.sfari.org/resource/sfari-base/. Further enquiries can be directed to the corresponding author.

## Footnotes

^1^ https://github.com/RiyaS-Verma/VariantCalling-Snakemake

^2^ http://www.pantherdb.org

^3^ https://david.ncifcrf.gov/home.jsp

^4^ https://metascape.org/gp/index.html#/main/step1

^5^descartes.brotmanbaty.org

^6^ https://www.ncbi.nlm.nih.gov/projects/gap/cgi-bin/study.cgi?study_id=phs000571.v6.p2

^7^ https://www.sfari.org/resource/sfari-base/
