# Supplementary material for: Mutations in genes related to myocyte contraction and ventricular septum development in non-syndromic tetralogy of Fallot
Source: Front Cardiovasc Med. 2023 Sep 28;10:1249605. doi: 10.3389/fcvm.2023.1249605 (PMC10569225; doi:10.3389/fcvm.2023.1249605)
Supplement: Supplementary file 4 [file Datasheet1.pdf]

*Supplementary Material*

# Mutations in genes related to myocyte contraction and ventricular septum development in non-syndromic tetralogy of Fallot

**Drayton C. Harvey, BA[1], Riya Verma, BS, MS[2], Brandon Sedaghat, BS, MS[3], Brooke E. Hjelm, PhD[4], Sarah U. Morton, MD, PhD[5], Jon G. Seidman, PhD[6], and S. Ram Kumar*, MD, PhD[7]**

Departments of [1]Pathology, [2]Stem Cell Biology and Regenerative Medicine, and [4]Translational Genomics, Keck School of Medicine of University of Southern California, Los Angeles, CA, USA

[3]Department of Medicine, Rosalind Franklin University School of Medicine and Science, Chicago, IL, USA

[5]Department of Pediatrics, Boston Children's Hospital, Boston, MA, USA

[6]Department of Genetics, Harvard Medical School, Boston, MA, USA

[7]Department of Surgery, University of Nebraska Medical Center, Omaha, NE, USA.

*** Correspondence:**
S. Ram Kumar, MD, PhD, FACS - Professor of Surgery

8200 Dodge St, Omaha, NE 68114

Tel: 402.955.4320 Email: rsubramanyan@unmc.edu

# 1 Supplementary Figures

**Supplemental Figure 1.** Tetralogy of Fallot cohort composition**. (A)** The cohort of TOF probands is mainly white, non-Hispanic/Latino (n=265), followed by white, Hispanic/Latino (n=36), Black, non-Hispanic/Latino (n=18), Asian, non-Hispanic/Latino (n=15), more than one race, non-Hispanic/Latino (n=12), more than one race, Hispanic/Latino (n=7), unknown race, Hispanic/Latino (n=4), Black, Hispanic/Latino (n=3), more than one race, unknown Hispanic/Latino (n=1), and unknown, Hispanic/Latino (n=1). A total of 13.81% of individuals in the cohort were identified as Hispanic/Latino (n=50). No individuals in the cohort were identified as American Indian, Alaska Native, Native Hawaiian or Pacific Islander. Race and ethnicity data was not collected on the SFARI cohort, instead ancestry was determined based on haplotypes identified through the HapMap project to be associated with certain ancestry groups. The composition of the SFARI cohort according to HapMap Phase III ancestry populations is as follows: 1.86% African ancestry in southwest United States, 30.75% Utah residents with northern and western European ancestry, 1.24% Han Chinese in Beijing, 1.24% Chinese in Denver, 1.86% Gujarati Indians in Houston, 1.24% Japanese in Tokyo, 1.55% Luhya in Webuye, Kenya, 9.63% Mexican ancestry in Los Angeles, 2.17% Maasai in Kinyawa, Kenya, 71.12% Toscana in Italy, 0.62% Yoruba in Ibadan, Nigeria and 4.04% unknown (some individuals have haplotypes for multiple populations and thus percentages do not add-up to 100%). **(B)** The number of probands eliminated at each stage of filtering is demonstrated from initial use of clinical diagnosis to implementation of FASTQ quality analysis, which ultimately reduced the initial 3,937 probands available in PCGC to 362 trios for variant calling and enrichment analysis. **(C)** While the SFARI and tetralogy of Fallot cohorts had similar *de novo* mutation rates, when comparing variants with a CADD of 20 or greater there was a significantly higher number of loss of function variants (p=0.05) and a significantly higher percentage of those variants were expressed in fetal cardiac tissue (p=0.05). * = $p \leq 0.05$

## 2 Supplementary Tables

**Supplemental Table 1. Variants called from Tetralogy of Fallot cohort.**

**Supplemental Table 2. Classifications of non-synonymous variants from Tetralogy of Fallot cohort.**

**Supplemental Table 3. Ingenuity Pathway Analysis enriched disease and function pathways and processes for Tetralogy of Fallot cohort.**
