# Supplementary figures and images for: Mutations in genes related to myocyte contraction and ventricular septum development in non-syndromic tetralogy of Fallot
Source: Front Cardiovasc Med. 2023 Sep 28;10:1249605. doi: 10.3389/fcvm.2023.1249605 (PMC10569225; doi:10.3389/fcvm.2023.1249605)

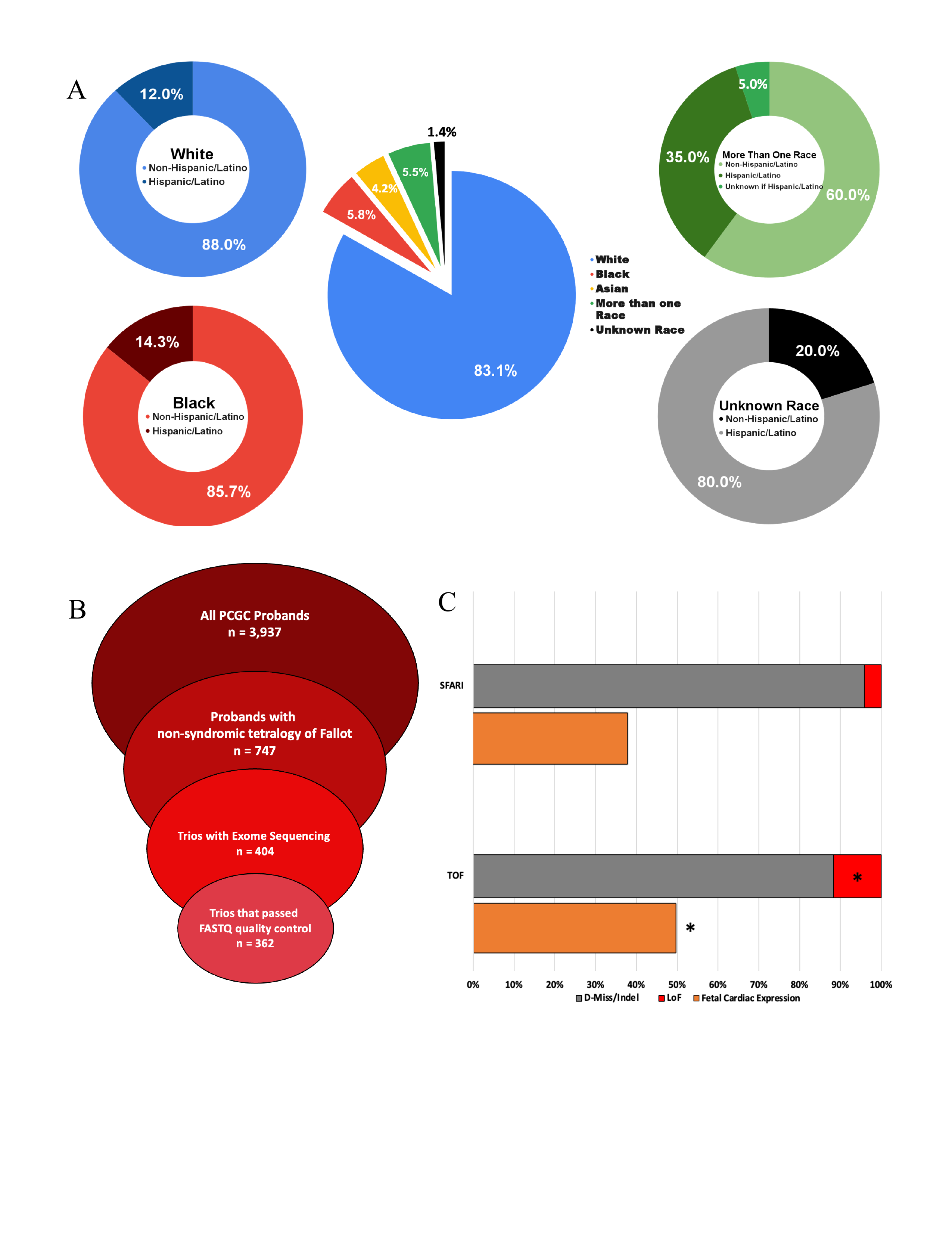

Supplement: Supplementary file 5 [file Image1.tiff]
